# Breastfeeding behaviours in women with obesity; associations with weight retention and the serum metabolome: a secondary analysis of UPBEAT

**DOI:** 10.1038/s41366-024-01576-6

**Published:** 2024-07-24

**Authors:** Kathryn V. Dalrymple, Annette L. Briley, Florence A. S. Tydeman, Paul T. Seed, Claire M. Singh, Angela C. Flynn, Sara L. White, Lucilla Poston, on behalf of the UPBEAT Consortium, Annette L. Briley, Paul T. Seed, Claire M. Singh, Angela C. Flynn, Lucilla Poston

**Affiliations:** 1 Department of Nutritional Sciences, School of Life Course and Population Sciences, King’s College London, London, UK; 2 Caring Futures Institute, CHNS, Flinders University, Adelaide, SA Australia; 3 Department of Women and Children’s Health, School of Life Course and Population Sciences, King’s College London, London, UK; 4 School of Population Health, Royal College of Surgeons in Ireland, Dublin, Ireland; 5 Guy’s and St Thomas’ NHS Foundation Trust, London, UK; 6 King’s College London, Guy’s and St Thomas’ NHS Foundation Trust, London, UK

**Keywords:** Nutrition, Lipidomics

## Abstract

**Background/Objectives:**

Maternal obesity is associated with a decreased intention and initiation of breastfeeding as well as a shortened duration of breastfeeding. This analysis was undertaken to identify breastfeeding behaviours, and relationships with maternal anthropometry and the serum metabolome at 6-months postpartum in an ethnically diverse cohort of women with obesity.

**Subjects/Methods:**

A cohort analysis of 715 women from the UK Pregnancies Better Eating and Activity Trial (UPBEAT); a multi-centre randomised controlled trial of an antenatal lifestyle intervention in women with obesity. Maternal data were collected in early pregnancy and included body mass index (BMI), socio-demographic characteristics and anthropometry. At 6-months postpartum, breastfeeding behaviours, anthropometry and 158 maternal metabolic measures from blood samples were recorded. Kaplan–Meier curves of breastfeeding duration were constructed and were stratified by obesity class (I: BMI 30.0–34.9 kg/m^2^, II: 35.0–39.9 kg/m^2^, III: ≥40.0 kg/m^2^). Relationships between breastfeeding behaviours, socio-demographic characteristics, the metabolome, and anthropometry were determined using regression analyses.

**Results:**

Eighty-two percent (591/715) of the cohort-initiated breastfeeding and at the 6-month follow-up 40% (283/715) were breastfeeding exclusively or partially. Duration of exclusive breastfeeding decreased with increasing BMI: Compared to BMI class I (mean 90.4 ± 64 days) the difference in mean for classes II and III were −15.8 days (95% confidence interval: −28.5, −3.1, *p* < 0.05) and −16.7 (95% CI: −32.0 to −1.35, *p* < 0.05), respectively. Compared to no breastfeeding, any breastfeeding at 6-months postpartum was associated with improvements in metabolites towards a healthier profile, reduced weight retention by −1.81 kg (95% CI −0.75, −2.88, p < 0.05 ) and reduced anthropometric measures, including mid-upper arm and hip circumferences. The breastfeeding related changes in anthropometry were not evident in women of Black ethnicity.

**Conclusions:**

Greater emphasis on enabling breastfeeding for women with obesity could improve duration, women’s weight management and metabolic health. The lack of breastfeeding related anthropometric effects in Black women requires further investigation.

**Clinical trial registry:**

ISRCTN reference 89971375.

## Introduction

The World Health Organization (WHO) recommends exclusive breastfeeding for the first 6 months of a child’s life for optimum infant development and health [1]. Despite this advice, exclusive breastfeeding rates in the UK at hospital discharge and 6–8 weeks postpartum remain low at ~70% [2] and~33%, respectively [3]. Some reports have shown that maternal obesity (BMI ≥ 30.0 kg/m^2^) is associated with a lower prevalence of breastfeeding compared with women of a healthy BMI (18.5–24.9 kg/m^2^) [4, 5], with up to 13% lower rates of initiation and 20% decreased likelihood of any breastfeeding by 6-months postpartum [4, 6], whereas others have reported no differences between initiation of breastfeeding and maternal BMI [7]. Despite wide cultural and international variation, maternal obesity has been associated with reduced breastfeeding rates, independent of country of study [8].

Barriers to breastfeeding in heterogeneous women with BMI, such as embarrassment, fear of pain and concerns about insufficient milk, are commonly reported [9, 10]. However, in women with obesity a range of factors further impact on breastfeeding initiation and duration, such as delayed lactogenesis, low prolactin and poor body confidence [6, 8, 11, 12]. These additional barriers faced by women with obesity are reflected in breastfeeding behaviours, including lower initiation and duration.

The metabolome, a descriptor for the small-molecule chemicals of body processes, responds to environmental and genomic interactions, and is increasingly utilised as a precision medicine tool with which to identify those at risk of cardiometabolic and other diseases. Whilst the benefit of breastfeeding on maternal weight and BMI is well recognised, the impact of breastfeeding on maternal metabolic health remains unclear [13]. Exploration of the metabolome in breastfeeding women may provide further insight into biological changes that occur during lactogenesis.

Participants were from the UK Pregnancy Better Eating and Activity Trial (UPBEAT), a multicentre randomised controlled trial of an antenatal lifestyle intervention. As the UPBEAT intervention did not affect breastfeeding initiation or duration, this data was treated as a cohort. In a secondary analysis, we have investigated breastfeeding initiation after birth and behaviours (exclusivity, mixed or no breastfeeding), anthropometry, postnatal weight retention (PPWR) and the impact breastfeeding had on the maternal metabolome in a population of women with obesity up to 6-months postpartum [14]. As UPBEAT participants were an ethnically diverse cohort, the influences of ethnicity have also been explored.

## Materials/subjects and methods

Between 2009–2014 UPBEAT recruited 1555 women (15^+0^–18^+6^ weeks’ gestation), with a BMI ≥ 30 kg/m^2^ (median 35.1 kg/m^2^ (IQR 32.8, 38.5)) from UK inner city settings, including, London, Glasgow, Newcastle, Sunderland, Bradford and Manchester. Participants were randomised to standard antenatal care or to a lifestyle intervention aimed at reducing dietary glycaemic load and increasing physical activity, superimposed on standard antenatal care. The primary aim of UPBEAT was to reduce the incidence of gestational diabetes (GDM) and large-for-gestational age infants. Sociodemographic, pregnancy information, anthropometric measurements and blood samples were obtained at study entry and at two further time points during pregnancy and at 6-months postpartum (2010–2015) [15]. All participants provided written informed consent. The NHS research ethics committee granted approval for all participating centres (UK integrated research application system, reference 09/H0802/5). Additional approvals were obtained through local Research and Development departments in each participating centre. UPBEAT was also registered with the ISRCTN reference 88971375).

At birth, pregnancy outcomes and mode of feeding were recorded. At 6-months postpartum, data on infant feeding intention in pregnancy, breastfeeding initiation at birth and duration were obtained. Additional data regarding infant weaning practices and the rationale for feeding choices for their infant were also recorded.

For the mother, weight was measured at the 6-month visit and the following circumferences and skinfold thicknesses were measured in triplicate and a mean obtained: neck, mid-arm, waist, hip (cm) and wrist (mm) using a tape measure. Skinfold thickness of triceps, biceps, suprailiac and subscapular were measured using Harpenden callipers and the sum of skinfolds was generated.

The primary maternal outcomes for this analysis were breastfeeding behaviours. For this study, these were defined as the percentage of all women who intended to breastfeed, the percentage of women who initiated breastfeeding (baby put to the breast on at least one occasion), the average duration of exclusive breastfeeding (infant received only breast milk, directly or expressed, and no other liquids or solids) and the percentage of any breastfeeding at 6-months postpartum.

Secondary maternal outcomes included the relationship between breastfeeding behaviours and obesity class (WHO obesity class I [BMI 30.0–34.9 kg/m^2^], II [BMI 35.0–39.9 kg/m^2^], III [≥40.0 kg/m^2^]), ethnicity, mode of birth, diagnosis of gestational diabetes and infant birthweight ≥4 kg. Maternal postnatal weight retention and anthropometry in relation to breastfeeding initiation and duration were also explored.

### Maternal metabolome

A total of 158 metabolites were evaluated using serum blood samples from 6-months postpartum. Targeted to multiple pathways relevant to obesity and insulin resistance, we used a high throughput NMR metabolomic platform (Nightingale Health Ltd, Finland). This platform accurately quantifies numerous lipid measures; lipoprotein particles include very low density (VLDL) subdivided into six subclasses (extremely large, very large, large, medium, small, very small), (Intermediate) IDL, (low) LDL subdivided into three subclasses (large, medium, small), and high (HDL) subdivided into four subclasses (very large, large, medium, small). The platform also elucidates the constituents within each lipoprotein particle type (triglyceride, total cholesterol, free cholesterol and cholesterol ester levels, and phospholipid concentrations). Fatty acids, amino acids, glycolysis related metabolites, ketone bodies and inflammatory markers are also measured.

### Statistical analyses

Missing data mechanism was assumed missing at random therefore a complete case analysis was used. All UPBEAT women who provided infant feeding data at the 6-month postpartum visit were included in the analysis. Chi-square and *t*-tests were used to investigate associations between breastfeeding behaviours and maternal variables. Descriptive statistics were used to identify the maternal rationales for choice of infant feeding, with percentages calculated for the proportion of women opting for each given reason when asked by the midwives during the follow up visits. Interval regression analysis with right sided censoring as not all mothers had stopped breastfeeding at the time of follow up was used to assess the duration of breastfeeding between obesity classes. Regression analyses were used to assess the difference between maternal variables for those who did and did not initiate breastfeeding.

For the serum metabolome, multivariable linear regression was applied for each metabolite with breastfeeding as the primary independent variable of interest, adjusted for the following confounders: age, BMI, parity, and intervention arm. The models for all women (*n* = 485) were also adjusted for ethnicity. All analytes were checked for normality and transformations were made as appropriate; analytes were then scaled and centred [16]. Results were presented as standard deviation (SD) differences between groups to allow for comparisons across multiple measured units. We present analysis for the entire cohort and, for comparison, for samples from women randomised to the control arm only. Metabolome analyses were also analysed by ethnicity for Black and White women. Women were excluded from the anthropometric and metabolomic analysis if they were pregnant at the 6-month follow-up visit. Statistical analyses were conducted using Stata (version 18) and RStudio version 3.5.2. *p* values ≤ 0.05 were considered statistically significant.

## Results

### Participants

**Fig. 1:**
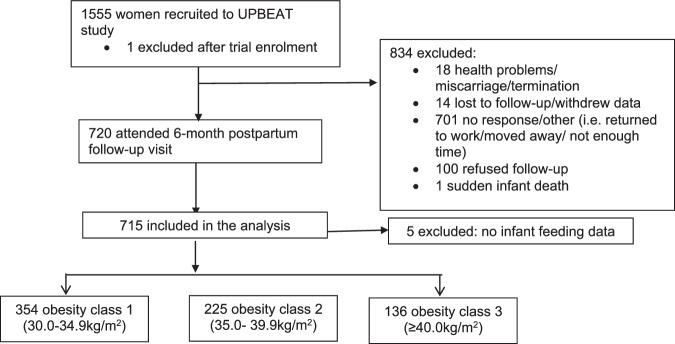
Summary diagram of recruitment process by maternal BMI at trial entry.

Data were available for 715/1555 (46.0%) UPBEAT participants who attended the 6-month postpartum follow-up visit. Of the 840 non-participants, 1 participant was excluded after randomisation, 19 pregnancies were affected by a major health problem, miscarriage or sudden infant death syndrome (SIDS); 100 declined participation; 701 either did not respond or were unable to participate as they had either, returned to full-time employment, living overseas or the child was not the primary responsibility of the woman. Five women had no infant feeding data recorded at the 6-month follow-up. Of those attending the 6-month postpartum visit with their child 354, 225 and 136 women were classified as BMI obesity classes I, II and III respectively at baseline (15^+0^ to 18^+6^ weeks gestation) (for study flow diagram see Fig. 1).

Compared to those women who did not return for the 6-month visit, the women who attended the 6-month follow-up were more likely to be of White ethnicity and less likely to be of Black or Asian ethnicity. Women were more likely to be nulliparous at study entry, less likely to be a smoker, more likely to have had GDM in pregnancy and more likely to have had an in-labour caesarean or an operative vaginal birth (Table 1).

**Table 1:** Demography of women included in analysis of breastfeeding behaviour compared to UPBEAT women who did not attend 6 months postnatal follow up visit.

	Included in analysis *n* = 715	Not included in analysis *n* = 839	Comparison
	Mean (SD)/*N* (%)		Beta-coefficient/Risk ratio/Odds ratio (95% CI)
Age (years)	31.2 (5.31)	29.9 (5.60)	1.32 (0.78–1.87)
BMI (kg/m^2^)	36.3 (4.90)	36.3 (4.66)	0.04 (−0.43–0.52)
Ethnic origin			
White	506 (71%)	467 (56%)	ref
Black	141 (20%)	260 (31%)	0.50 (0.39–0.63)***
Asian	5 (3%)	70 (8%)	0.33 (0.20–0.53)***
Other	43 (6%)	42 (5%)	0.94 (0.61–1.47)
Nulliparous	363 (51%)	311 (37%)	1.75 (1.43–2.14)***
Current smoker	27 (4%)	81 (10%)	0.37 (0.23–0.57)***
Index of multiple deprivation			
1 (least deprived)	35 (5%)	30 (4%)	1.51 (0.91–2.51)
2	49 (7%)	54 (6%)	1.17 (0.77–1.78)
3	76 (11%)	101 (12%)	0.97 (0.60–1.36)
4	261 (37%)	272 (33%)	1.24 (0.98–1.56)
5 (most deprived)	292 (41%)	378 (45%)	ref
Pregnancy outcomes			
Diagnosis of GDM^a^	197/699 (28%)	141/606 (23%)	1.29 (1.00–1.66)*
Gestation at birth ≤37 weeks	32/715 (4.5%)	67/805 (8.3%)	0.52 (0.33–0.79)
Unassisted vaginal birth	345/715 (48%)	453/805 (56%)	ref
Operative vaginal birth	93/715 (13%)	85/805 (10%)	1.43 (1.04–1.99)*
Pre-labour caesarean section	144/715 (20%)	151/805 (19%)	1.25 (0.96–1.63)
In labour caesarean section	133/715 (18%)	116/805 (14%)	1.50 (1.13–2.00)**
Birthweight ≥4 kg	101/715 (14%)	109/805 (13%)	1.05 (0.78–1.41)

*BMI* body mass index, *CI* confidence interval, *GDM* gestational diabetes, *SD* standard deviation.

*p < 0.05; **p < 0.01; ***p < 0.001.

^a^OGTT results not available for all participants therefore denominator noted here.

### Effect of intervention

There was no significant difference in breastfeeding intention, initiation or exclusive or partial breastfeeding at 6 months by UPBEAT group allocation (Supplementary Table 1). Therefore, data for intervention and control arms of the trial were combined and the participants treated as a cohort.

### Breastfeeding behaviours

Table 2 summarises breastfeeding intention and practices by maternal BMI class at trial entry for those who completed the 6-month follow up visit. Overall, 76.3% of women stated antenatally that they intended to breastfeed, and 82.7% put their baby to the breast on at least one occasion. For those women who initiated breastfeeding, the percentage of any breastfeeding at 6-months postpartum decreased in BMI class III compared to classes I and II (class 1, 51.7%, II, 48.2% and III 30.7%, *p* < 0.05). The percentage of those using formula milk at the 6-month follow-up increased with BMI class, 75.0%, 80.0% and 84.0%, respectively.

**Table 2:** Infant feeding outcomes recorded at the 6-month follow-up, stratified by maternal BMI class at trial entry.

	BMI Class I (30.0–34.9 kg/m^2^)	BMI Class II (35.0–39.9 kg/m^2^)	BMI Class III (≥40.0 kg/m^2^)
*N* (%)/Mean (standard deviation)			
Intended to breastfeed	267/354 (75.4%)	178/225 (79.1%)	101/136 (74.3%)
Initiated breastfeeding at birth	294/354 (83.1%)	189/225 (84.0%)	108/136 (79.4%)
6-month breastfeeding status of those who initiated breastfeeding			
Age of infant at 6-month follow-up	5.9 (0.86)	5.8 (0.87)	6.0 (1.01)
Exclusive breastfeeding	11/294 (3.7%)	7/189 (3.7%)	1/108 (0.9%)
Formula milk only	8/294 (2.7%)	6/189 (3.2%)	0/108 (0%)
Breastfeeding + other fluids	4/294 (1.3%)	4/189 (2.1%)	5/108 (4.6%)
Breastfeeding + solids ± other fluids	73/294 (24.8%)	41/189 (21.6%)	16/108 (14.8%)*
Formula + solids ± other fluids	134/294 (45.6%)	92/189 (48.7%)	68/108 (63.0%)*
Breastfeeding + Formula milk + solids ± other fluids	64/294 (21.7%)	39/189 (20.6%)	18/108 (16.7%)*
Any breastfeeding at 6 months	152/294 (51.7%)	91/189 (48.2%)	40/108 (30.7%)*
All women			
Formula feeding at 6 months	266/354 (75.0%)	203/255 (80.0%)	114/136 (84.0%)
Introduced solids at 6 months	329/354 (92.9%)	201/225 (89.3%)	128/136 (94.1%)
Mean duration of exclusive breastfeeding (days)	90.4 (64.0)	74.6 (66.3)	73.7 (65.1)
Difference in mean (95%CI) vs. class I		−15.8 (−28.5, −3.1)*	−16.7 (−32.0, −1.35)*
Never breastfed	60 (17.0%)	36 (16.0%)	28 (20.7%)

Other fluids defined as drinks such as water or juice.

*p < 0.05.

**Fig. 2:**
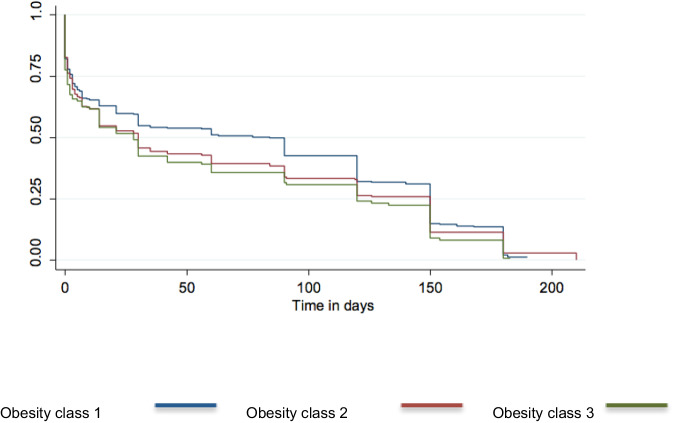
Kaplan–Meier survival curve for duration of exclusive breastfeeding from birth until cessation in women in each obesity class.

Figure 2 shows a Kaplan–Meier survival curve for breastfeeding duration according to BMI category. Interval regression analysis showed duration of exclusive breastfeeding in women with class I obesity was 90.4 days compared to 74.6 days and 73.7 days in those with class II and class III obesity, respectively; mean differences were: class II −15.8 (95% confidence interval (CI) −28.5 to −3.1), *p* < 0.01) and class III −16.7 (95% CI −32.0 to −1.35), *p* < 0.05), compared to class I (Table 2). More women with obesity class I compared with class II and III were more likely offering breastfeeding in combination with solids and other fluids (e.g., water) at 6-months postpartum (24.8, 21.6 and 14.8%) (Table 2). 92% of the cohort had introduced solids by the 6-month follow-up.

Breastfeeding behaviours demonstrated associations with educational attainment; a higher education attainment was associated with a likelihood of partial or exclusive breastfeed at 6-months postpartum. Maternal age ≥30 years and cohabitation were associated with a higher percentage of breastfeeding initiation and duration (Supplementary Table 2). Whereas being of White ethnicity and smoking were associated with lower rates of breastfeeding initiation and duration.

Table 3 presents the birth outcomes stratified by BMI class. There was no statistical difference in the relationship between initiation of breastfeeding and maternal obesity category (Table 3). There were no differences between mode of birth or infant birthweight ≥4 kg when stratified by maternal BMI. Diagnosis of GDM was significantly more common in BMI classes II and III, compared to class I (Table 3).

**Table 3:** Initiation of breastfeeding and pregnancy outcomes according to BMI class.

	BMI Class I (30.0–34.9 kg/m^2^)	BMI Class II (35.0–39.9 kg/m^2^)	BMI Class III (≥40.0 kg/m^2^)	Class 2 compared to class 1	Class 3 compared to class 1
		*N* (%)		Odds ratio (95% CI)	
Initiation of breastfeeding	294/354 (83.1%)	189/225 (84.0%)	108/136 (79.4%)	1.07 (0.68–1.68)	0.79 (0.48–1.30)
Parity	176/354 (50%)	120/354 (53%)	67 (49%)	1.15 (0.83, 1.62)	0.98 (0.66, 1.46)
Unassisted vaginal	176/354 (50%)	112/225 (50%)	57/136 (42%)	ref	ref
Operative vaginal	44/354 (12%)	33/225 (15%)	16/136 (11%)	1.18 (0.71–1.96)	1.12 (0.59–2.14)
Pre-labour caesarean	75/354 (21%)	37/225 (16%)	32/136 (23%)	0.78 (0.49–1.23)	1.32 (0.79–2.19)
Caesarean in labour	59/354 (17%)	43/225 (19%)	31/136 (23%)	1.14 (0.72–1.81)	1.62 (0.96–2.75)
GDM diagnosis	77/345 (22%)	75/218 (34%)	45/136 (33%)	1.82 (1.25–2.66)**	1.11 (1.11–2.66)*
Infant birthweight ≥4 kg	54/354 (15%)	30/221 (13%)	17/136 (13%)	0.85 (0.53–1.38)	0.79 (0.44–1.43)

Data presented as number of women/total (%).

*BMI* body mass index, *CI* confidence interval, *GDM* gestational diabetes.

*p < 0.05; **p < 0.01.

### Weight and anthropometric measures

Supplementary Table 3 summarises the data stratified by any vs. no breastfeeding, at 6-months postpartum. Women who were offering any breast milk at 6 months (*n* = 283) weighed 1.12 kg less than their pre-pregnancy weight. Whereas women who were not breastfeeding at 6 months demonstrated weight retention of 0.70 kg (mean difference of 1.81 kg (95% CI 0.75, 2.88) *p* < 0.01). Changes from baseline to 6 months in neck, mid-arm, wrist and hip circumferences were also significantly lower in women who were fully or partially breastfeeding at 6 months compared to those women who were not breastfeeding. There were no statistically significant associations between maternal skinfold thicknesses and breastfeeding behaviours (Supplementary Table 3).

Subgroup analysis between women of Black and White ethnicity showed that changes in weight retention and circumferences associated with breastfeeding apparent in the White women were not evident in women of Black African or Black Afro-Caribbean ethnicity (Supplementary Table 4). Black women who were breastfeeding at the 6-month follow-up had an average postpartum weight retention of 3.35 kg (95% CI 1.39, 5.30) compared to White women. Similar observations were apparent in mid-upper arm [0.93 (0.36, 1.51)] and hip circumferences [3.59 (2.00, 5.19)]. However, women of Black ethnicity were more likely to initiate breastfeeding (95% vs. 78%) and be breastfeeding at the 6-month follow-up (62% vs. 32%) compared to women of White ethnicity (Supplementary Table 2).

### Metabolome

**Fig. 3:**
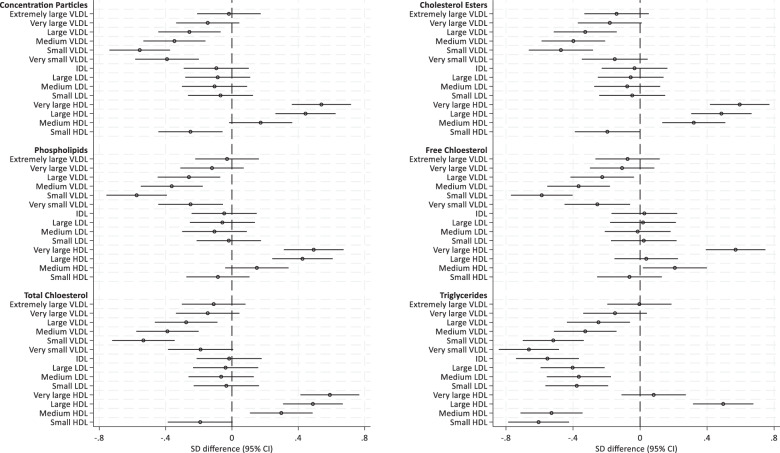
Standard deviation difference in lipoprotein particle concentration and subclass constituents between breastfeeding and non-breastfeeding UPBEAT womenat 6 months postpartum (*n* = 485). The right-hand side of the *x*-axis represents positive associations with breastfeeding (fully or mixed) at 6 months, compared to non-breastfeeding women, and negative associations to the left-hand side.

**Fig. 4:**
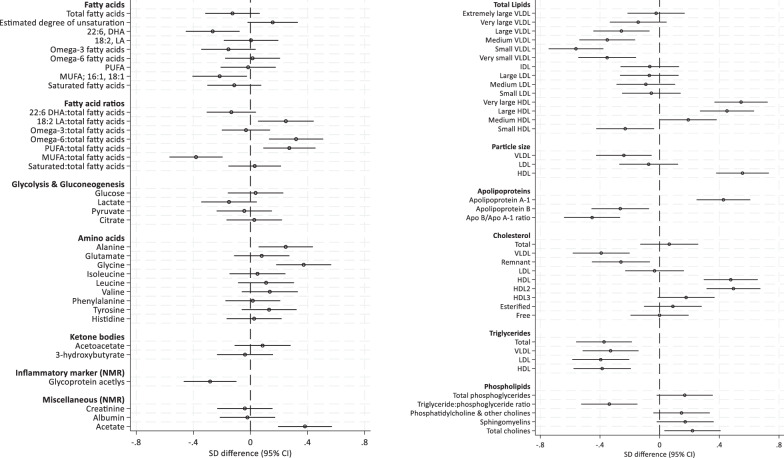
Standard deviation difference for fatty acids, amino acids, glycaemic and other markers, as well as lipoprotein particle groups between breastfeeding and non-breastfeeding UPBEAT women at 6 months postpartum (*n* = 485). The right-hand side of the x-axis represents positive associations with breastfeeding (fully or mixed) at 6 months, compared to non-breastfeeding women, and negative associations to the left-hand side.

There were no significant differences in the metabolome at 6-months postpartum between women randomised to the control and intervention arms following adjustment for age, ethnicity and parity, and the data were therefore treated as a cohort. Exclusive or partial breastfeeding vs. no breastfeeding at 6-months postpartum was associated with marked changes in the NMR metabolome (Figs. 3 and 4). Breastfeeding was associated with a reduction in some metabolites and an increase in others. A marked reduction in total triglycerides was observed (Fig. 4b), reflecting a reduction within multiple subclasses of VLDL, LDL and HDL lipoprotein particles, and within the IDL lipoprotein subclass (Fig. 3b). VLDL particle 13-1 was smaller (Fig. 4b), and there were lower total lipids in VLDL (Very large, large, medium, small and very small) subclasses which was attributable to lower VLDL triglycerides, cholesterol, and phospholipids (Fig. 3a, b). HDL particle size was higher and there were higher total lipids in HDL (very large and large) subclasses (Fig. 4b) which was attributable to greater total cholesterol and phospholipid content (Fig. 3a). Apolipoprotein A-1 concentration was higher and Apolipoprotein B and the ApoA/ApoB ratio were lower (Fig. 4b). When expressed as proportions of total fatty acids, polyunsaturated fatty acids were increased (linoleic, omega-6 and PUFA), there was a decrease in mono-unsaturated fatty acids (Fig. 4a). In addition, glycoprotein acetyls, an inflammatory marker, was reduced, whereas acetate and the amino acids alanine and glycine were increased (Fig. 4a) in the breastfeeding group compared to those who did not breastfeed.

When considered according to ethnic group, a similar metabolic profile to the whole group was observed in the White women who were breastfeeding at 6-months postpartum (Supplementary Figs. 1 and 2). However, although numbers were smaller there were a number of notable differences seen in the metabolome of Black breastfeeding mothers compared to White mothers; IDL and LDL particle size, concentration and content, were little impacted by breastfeeding in White women (apart from triglyceride content), there was a tendency for these to be lower in breastfeeding women of Black ethnicity, likely as a result of lower phospholipid and total cholesterol content in these particles (Supplementary Fig. 1a, b). Reductions in total fatty acids including polyunsaturated, monounsaturated and saturated fatty acids appeared more marked in women of Black ethnicity who breastfed (Supplementary Fig. 2a). Supplementary Figs. 3 and 4 demonstrate that the metabolic profile comparing breastfeeding and non-breastfeeding women in the control arm only (*n* = 253), was similar to that in the entire cohort (*n* = 485).

### Reasons for maternal choices regarding infant feeding

Of the 124 women who did not initiate breastfeeding, 64% reported “never planned to”, 13% gave reasons including “difficulty in establishing lactation”, “feeding issues with a previous child” and “inverted nipples”. A further 5% reported being “advised not to breastfeed”. Of those who initiated breastfeeding but stopped before their child was 6-months old, the most common reason was “perceived inadequate milk supply” (23%); others were “discomfort” (7%) and “convenience” (5%). Thirty nine percent of women reported “other” reasons for cessation including: infant tongue tie, difficulty in ‘latching on’, needing to return to work and partner/family members wanting to feed the baby. Of the 290 women who maintained some breastfeeding at 6-months, the most cited reasons for continuing were that breast milk is the “best nutrition for baby” (74%), “convenience” (55%), “enjoyment” (47%), “cheaper” (37%) and “maternal weight loss” (22%).

## Discussion

This study reports original observations on breastfeeding behaviours and the metabolome in an ethnically diverse cohort of women with obesity. The findings also strengthen the case for additional support and strategies to enable women with obesity to breastfeed for a longer duration. We have described the relationship between severity of obesity and breastfeeding and found that women with a higher BMI had a shorter exclusive breastfeeding duration of 17 days compared with women from a lower obesity class. We have also described the previously recognised relationships between breastfeeding and reduction of postpartum weight and measures of anthropometry, although these positive changes in anthropometry were not observed amongst women of Black ethnicity, despite Black women being more likely to initiate and continue to breastfeed until 6-months postpartum, compared to other ethnic groups.

Breastfeeding duration has previously been identified as an important determinant of maternal weight loss postpartum [17, 18], with previous reports documenting the largest reduction in weight amongst women of heterogeneous BMI breastfeeding at 6-months and beyond [17, 19]. Compared to no breastfeeding, we have reported a −1.8 kg difference in those women who were giving any breastfeeding at 6-months postpartum. Although modest, our study confirms that breastfeeding duration is equally relevant to weight loss in women with obesity; and indeed, to reduce adiposity, providing further evidence for healthcare professionals to support women with obesity to breastfeed, and to encourage longer durations of breastfeeding to aid postpartum weight loss. An ongoing randomised controlled trial in Columbia is investigating the impact of breastfeeding counselling on breastfeeding prevalence and postpartum weight loss in women with a BMI > 24.9 kg/m^2^, which may contribute to an evidence based intervention for encouraging women with a higher BMI to breastfeed [20].

In contrast to the present study, a study from the USA with smaller sample size (*n* = 37) reported that postnatal weight retention in women with obesity was associated with increased energy intake, independent of breastfeeding, eating behaviours and metabolic biomarkers [21]. The authors investigated body composition, diet and activity from early pregnancy until 12 months postpartum and stratified results by PPWR vs. postpartum weight loss. Duration of breastfeeding was similar in both groups (30 ± 5 vs. 29 ± 6 weeks), indicating that higher energy intake could override the role of breastfeeding in postpartum weight loss.

We found that intention to, and initiation of, breastfeeding in the study cohort of women with obesity was higher than reported in a general UK population [2, 3]. Contributary factors may include participation in a clinical trial, or changes in local midwifery and health visitor practice to support breastfeeding in line with the UK Baby Friendly Initiative [22]. The follow up rate of 46% of the original trial participants could also reflect selection bias, with findings less generalisable to the whole study cohort, although those choosing not to take part at 6-months postpartum had similar characteristics at baseline to the participants included in this analysis.

We found that increasing BMI class was associated with decreased duration of breastfeeding. A retrospective cohort study of women from the USA reported a similar relationship, although with different BMI classification; 18.5–24.9 kg/m^2^; 30.0–39.9 kg/m^2^; 40.0–49.9 kg/m^2^; ≥50.0 kg/m^2^ [23]. The authors found that overall breastfeeding rates were low (32%) with no data on continued breastfeeding beyond hospital discharge, a strength of the present study.

Previously reported reasons for decreased duration of breast feeding in women with obesity have included reduced maternal confidence to breastfeed associated with larger breasts [8, 11] and delayed lactogenesis II [24]. Once initiated, milk supply may be impacted by hormonal imbalance [8, 25] or through consequences of the mother’s perceived body image [12].

We report here reasons given by the study participants for non-initiation and shorter duration of breastfeeding. These indicated that family and healthcare staff support are important in facilitating breastfeeding in women with obesity. To achieve this would require relevant healthcare staff in acute and primary care settings to acquire appropriate competencies and skills, with inherent cost implications. Insufficient regulations of the marketing of breastmilk substitute as well as food insecurity also undermine breastfeeding prevalence [26, 27, 28]. Also, more research is required to explore women’s perceptions of inadequate milk supply and to identify why some of the participants, and indeed the wider population of women, do not consider breastfeeding.

A 2017 narrative review suggested that interventions aimed at breastfeeding women will not be successful unless there is protection, promotion, and support at a population health level, along with increased investment in health services to support women to breastfeed [29]. Furthermore, open responses from the participants indicated that at 6-months postpartum many had returned to paid employment, or were imminently planning to, a common reason for early weaning and cessation of breastfeeding. Comments from women on reasons for not initiating breastfeeding, or for stopping early, highlighted several areas for further research to better comprehend the complexity surrounding maternal breastfeeding behaviours.

Our study was also supported by a biological ‘read out’ of metabolic health through the NMR metabolome at 6-months postpartum, until which time the WHO recommend exclusive breastfeeding. To our knowledge changes in the metabolome in women with obesity continuing to breastfeed at 6-months postpartum, either partially or exclusively, have not previously been reported and may provide insight into the mechanisms of weight loss associated with breastfeeding [19, 30]. This could contribute to the protective effect of breastfeeding against progression to diabetes after gestational diabetes [31, 32]. There were numerous indications of metabolic health, when compared with non-breast feeding women, including a reduction in atherogenic VLDL particles and triglycerides across lipoprotein particles, and an increase in anti-atherogenic larger HDL particles, including constituent HDL cholesterol and phospholipids. In addition, continuation of breastfeeding was associated with higher polyunsaturated fatty acids and lower mono-unsaturated fatty acids. The majority of fats in breastmilk comprise triglycerides, synthesised in the mammary glands of the breast from de-novo lipogenesis of breast fat [33, 34] and intact triglycerides are not directly transported from the circulation into breast milk [35]. The fall in maternal plasma triglycerides observed in association with breastfeeding likely reflects increased mobilisation of maternal fat stores and enhanced metabolism to fatty acids that would contribute to generation of energy to meet the demands of breastfeeding. Mechanistically, it has been proposed that the stimulation of prolactin during lactation would lead to a fall in maternal oestrogens which, in turn, would stimulate lipolysis [18]. An increase in the proteogenic amino acid glycine could be advantageous to maternal health. Our findings are consistent with a targeted mass spectrometry metabolome undertaken 6–8 weeks postpartum in a cohort of individuals with normoglycaemia but with previous GDM, where lactation intensity was associated with lower triglycerides (and diglycerides) and higher phospholipids [36]; indeed they suggest that downregulation of triglycerides/diglyceride lipogenesis during lactation is directly associated with formation of phospho- and sphingolipids through the CEPT1 gene. In addition, the ‘healthy’ metabolic profile seen here mirrors that seen during pregnancy in individuals with obesity who were normoglycaemic compared to those who had GDM [37] and may reflect a comparatively insulin-sensitive state in those who are breastfeeding. As we had previously reported a beneficial effect of the intervention on the maternal metabolic profile between 16 and 36 weeks of gestation [38], to exclude any residual effect of the intervention postpartum we also analysed the metabolome in women in the control arm, which demonstrated similar differences in the profile between the breastfeeding and non-breastfeeding mothers as that of the metabolome from the whole cohort.

The lack of effect of breastfeeding on weight and measures of adiposity in women of Black African or Black Afro-Caribbean ethnicity compared with women of White ethnicity is a novel observation. There may be fundamental differences of genetic origin in fat metabolism, or differences in postpartum physical activity, and diet. We have previously reported [39] the longitudinal dietary trajectories in the UPBEAT women across pregnancy and up to 3-years postpartum. Amongst the women who had a high adherence to an African/Caribbean dietary pattern, we observed a dietary rebound for those women who followed a high adherence to the African/Caribbean trajectory at 6-months postpartum. This may reflect food insecurity, sociocultural drivers of diet such as social support or family food preferences and may contribute to the null findings for postpartum weight loss for women of Black ethnicity. Differences in the metabolome observed between White and Black women who breastfeed are novel. These may be driven by a modification in their diets [39], or implicated by the lower sample of Black women in the study; these findings need to be evaluated in a larger cohort.

### Strengths and limitations

Strengths of the study include the rich UPBEAT dataset which provided comprehensive information on breastfeeding and anthropometry outcomes at 6-months postpartum in an ethnically diverse cohort. To our knowledge, this study is the first to report metabolomic changes associated with breastfeeding in a cohort of women with obesity. The main limitation is the observational study design, which is subject to residual confounding and potential overestimation of reported effects [40]. Although the breastfeeding outcomes are reported in detail, these outcomes are self-reported by the mother which may be subject to recall bias [41]. Furthermore, while the findings are generalisable amongst women with obesity, they may not be directly generalisable to the general population of women with a heterogeneous BMI.

## Conclusion

Given the prevalence of obesity in women of reproductive age, strategies to support and enable women with a BMI ≥ 30.0 kg/m^2^ to initiate and continue to breastfeed are required to improve long-term maternal health outcomes. This study supports strategies to encourage, support and enable all women to continue breastfeeding for at least 6 months, according to the WHO recommendation, and to overcome barriers associated with breastfeeding. The initiation and duration of breastfeeding requires collective societal approaches, including support from family members, healthcare professionals, as well as government action to enable and support breastfeeding, such as the development of actionable policies which promote breastfeeding and reduce the misleading advertising and marketing of breastmilk substitutes [42]. Further exploration into the null findings for postpartum weight loss in women of Black ethnicity are required.

## Supplementary information

Supplementary file

## Data Availability

The datasets generated during and analysed during the current study are available from the corresponding author on reasonable request pending application (via a research application form) and approval by the UPBEAT Consortium.
