# Supplementary material for: Breastfeeding behaviours in women with obesity; associations with weight retention and the serum metabolome: a secondary analysis of UPBEAT
Source: Int J Obes (Lond). 2024 Jul 24;48(10):1472–80. doi: 10.1038/s41366-024-01576-6 (PMC11420090; doi:10.1038/s41366-024-01576-6)
Supplement: Supplementary file 1 — Supplementary file [file 41366_2024_1576_MOESM1_ESM.docx]

| **Supplementary Table 1:** Breast feeding behaviour by randomization arm of UPBEAT | | | |
| --- | --- | --- | --- |
|  | **Intended to breastfeed** | **breastfeed initiated** | **Any breastfeed at 6 months** |
| **UPBEAT randomisation** | | | |
| Intervention | 277/354 (78%) | 300/354 (85%) | 136/354 (38%) |
| Control | 269/361 (75%) | 291/361 (81%) | 147/361 (40%) |

| **Supplementary Table 2**. Relationships between maternal breastfeeding practices at 6 months follow up and baseline demography | | | | | |
| --- | --- | --- | --- | --- | --- |
|  | **Intended to BF**  **N=546** | **BF initiated**  **N=591** | **Any BF at 6 months**  **N=283** | **Exclusive BF at 6 months**  **N=19** | **Introduction of solids at 6-month visit**  **N=658** |
| ≥30 years | 363/457 (79%) | 395/457 (86%) | 208/457 (45%) | 16/457 (3%) | 413/457 (90%) |
| **Ethnicity** |  |  |  |  |  |
| White | 377/506 (74%) | 395/506 (78%) | 163/506 (32%) | 10/506 (2%) | 470/506 (93%) |
| Black | 111/141 (79%) | 134/141 (95%) | 88/141 (62%) | 7/141 (5%) | 126/141 (89%) |
| Asian | 20/25 (80%) | 22/25 (88%) | 13/25 (52%) | 1/25 (4%) | 22/25 (88%) |
| Other | 38/43 (88%) | 40/43 (93%) | 22/43 (51%) | 1/43 (2%) | 40/43 (93%) |
| **Highest education attainment** | |  |  |  |  |
| None | 8/15 (53%) | 10/15 (67%) | 6/15 (40%) | 0/15 (0%) | 13/15 (87%) |
| GCSE | 60/91 (66%) | 65/91 (71%) | 25/91 (27%) | 2/91 (2%) | 81/91 (89%) |
| A-Level | 85/112 (76%) | 96/112 (86%) | 38/112 (34%) | 2/112 (2%) | 105/112 (94%) |
| Vocational | 108/169 (64%) | 111/169 (66%) | 36/169 (21%) | 3/169 (2%) | 160/169 (95%) |
| First degree | 182/215 (85%) | 200/215 (93%) | 107/215 (50%) | 10/215 (5%) | 190/215 (88%) |
| Higher degree | 103/113 (91%) | 109/113 (96%) | 71/113 (63%) | 2/113 (2%) | 109/113 (96%) |
| **Index of multiple deprivation** | |  |  |  |  |
| 5 (most deprived) | 219/292 (75%) | 237/292 (81%) | 112/292 (38%) | 11/292 (4%) | 263/292 (90%) |
| Primiparous^1^ | 293/363 (81%) | 313/363 (86%) | 144/363 (40%) | 9/345 (2%) | 338/345 (93%) |
| **Living arrangements** |  |  |  |  |  |
| Cohabiting^1^ | 463/588 (79%) | 494/588 (84%) | 244/588 (42%) | 17/588 (3%) | 542/588 (92%) |
| Current smoker | 16/27 (60%) | 16/27 (60%) | 4/27 (15%) | 1/27 (4%) | 24/27 (89%) |

^1^Primip: index pregnancy =first pregnancy.

^2^Cohabiting: Married or living with partner. Abbreviations: BF: breastfeeding

| **Supplementary table 3**: Maternal weight and anthropometry, and change in maternal weight and anthropometry between baseline (15^+0^ to 18^+6^ weeks’ gestation) and 6-months postpartum according to breast feeding practices at 6 months postpartum | | | | |
| --- | --- | --- | --- | --- |
|  | **Any BF n= 281** | **No BF n=424** | **Comparison n=705** | **p** |
|  | **Mean (SD)** | | **Difference in mean (95% CI)** |  |
| Baseline weight (kg) | 96.6 (14.5) | 99.7 (16.3) | 3.08 (0.74, 5.43) | <0.01 |
| Weight at 6 months (kg) | 95.7 (16.0) | 100.3 (16.6) | 4.59 (2.10, 7.09) | <0.001 |
| Change in weight (kg) | -1.12 (7.4) | 0.70 (6.8) | 1.81 (0.75, 2.88) | <0.001 |
| **Anthropometry at 6-months postpartum according to breast feeding behaviour** | | | | |
| **Circumferences** **(cm)** | | | | |
| Neck | 36.6 (2.5) | 37.1 (2.7) | 0.56 (0.15, 0.96) | <0.01 |
| Waist | 104.4 (11.0) | 107.8 (11.9) | 3.36 (1.60, 5.12) | <0.0001 |
| Midarm | 36.6 (4.4) | 37.8 (4.2) | 1.14 (0.49, 1.79) | <0.0001 |
| Wrist (mm) | 171.5 (15.4) | 174.0 (14.7) | 2.59 (0.31, 4.87) | 0.03 |
| Hip | 120.8 (10.9) | 124.0 (11.5) | 3.16 (1.44, 4.88) | <0.001 |
| **Skinfold thicknesses (mm)** | | | | |
| Sum of skinfolds | 120.3 (28.4) | 122.6 (27.0) | 2.34 (-1.90, 6.55) | 0.276 |
| **Changes in maternal anthropometry from baseline to 6-months postpartum by breast feeding behaviour** | | | | |
| **Circumferences** **(cm)** | | | | |
| Neck | 0.1 (1.6) | 0.6 (1.9) | 0.46 (0.19, 0.73) | <0.001 |
| Waist | -1.13 (7.5) | -0.93 (7.3) | 0.21 (-0.92, 1.32) | 0.718 |
| Midarm | 0.03 (2.3) | 0.91 (2.5) | 0.87 (0.37, 1.25) | <0.001 |
| Wrist (mm) | -1.21 (11.8) | 1.56 (13.1) | 2.77 (0.85, 4.70) | <0.01 |
| Hip | -0.84 (6.9) | 0.47 (6.9) | 1.30 (0.24, 2.35) | 0.012 |
| **Skinfold thicknesses (mm)** | | | | |
| Sum of skinfolds | -2.67 (27.9) | -0.48 (25.7) | 2.20 (-1.89 to 6.28) | 0.292 |
| Abbreviations: BF: breastfeeding, CI: confidence interval, SD: standard deviation  N=10 women were excluded as they were pregnant at the 6-month follow-up | | | | |

| **Supplementary Table 4**: Anthropometric changes associated with any breastfeeding comparing Black vs White women at baseline (15^+0^ –18^+6^) and 6-months postpartum | | |
| --- | --- | --- |
|  |  | |
|  | Any breastfeeding (n= 246) | No breastfeeding (n=391) |
| Difference in mean (95% CI) | | |
| Baseline weight (kg) | 1.15 (-2.71, 5.03) | 0.08 (-4.65, 4.81) |
| Weight at 6 months (kg) | 4.57 (0.30, 8.83) ^*^ | 0.84 (-4.11, 5.79) |
| Change in weight (kg) | 3.35 (1.39, 5.30) ^**^ | 0.25 (-1.65, 2.16) |
| **Anthropometry at 6-months postpartum** | | |
| **Circumferences** **(cm)** |  |  |
| Neck | 1.39 (0.75, 2.03) ^***^ | 0.72 (-0.10, 1.54) |
| Waist | 2.99 (0.05, 5.93) ^*^ | -0.66 (-4.19, 2.86) |
| Midarm | 2.53 (1.43, 3.63) ^***^ | 0.62 (-0.64, 1.88) |
| Wrist (mm) | 0.90 (-3.30, 5.10) | 3.39 (-1.00, 7.78) |
| Hip | 2.69 (-0.16, 5.54) | -2.19 (-5.64, 1.25) |
| **Skinfold thicknesses (mm)** | | |
| Sum of skinfolds | 20.9 (13.59, 28.13) ^***^ | 8.63 (0.72, 16.54) ^*^ |
| **Changes in maternal anthropometry from baseline to 6-months postpartum** | | |
| **Circumferences** **(cm)** |  |  |
| Neck | 0.33 (-0.09, 0.75) | 0.16 (-0.39, 0.70) |
| Waist | 0.53 (-1.52, 2.57) | -1.53 (-3.66, 0.60) |
| Midarm | 0.93 (0.36, 1.51) ^*^ | -0.073 (-0.82, 0.66) |
| Wrist (mm) | 2.09 (-1.17, 5.36) | 3.32 (-0.58, 7.24) |
| Hip | 3.59 (2.00, 5.19) ^***^ | -0.39 (-2.42, 1.64) |
| **Skinfold thicknesses (mm)** | | |
| Sum of skinfolds | 4.60 (-3.02, 12.23) | -3.36 (-10.88, 4.17) |
| ^*^ p<0.05, ^**^ p<0.01 ^***^ p<0.001, Abbreviations: CI: confidence interval  N=10 women were excluded as they were pregnant at the 6-month follow-up visit. | | |

^^

Supplementary Figure 1a: Standard deviation differences in lipoprotein particle concentration and subclass constituents between breastfeeding and non-breastfeeding UPBEAT women at 6 months postpartum [for all women=485, those of White ethnicity (n=356) and those of Black ethnicity (n=83)]. The right-hand side of the x-axis represents positive associations with breastfeeding (fully or mixed) at 6 months, compared to non-breastfeeding women, and negative associations to the left-hand side. Multivariable linear regression was applied for each metabolite with breastfeeding as the primary independent variable of interest, adjusted for the following confounders: age, BMI, parity, and intervention. The models for all women (n=485) were also adjusted for ethnicity.

Supplementary Figure 1b: Standard deviation differences in lipoprotein subclass constituents between breastfeeding and non-breastfeeding UPBEAT women at 6 months postpartum [for all women=485, those of White ethnicity (n=356) and those of Black ethnicity (n=83)]. The right-hand side of the x-axis represents positive associations with breastfeeding (fully or mixed) at 6 months, compared to non-breastfeeding women, and negative associations to the left-hand side. Multivariable linear regression was applied for each metabolite with breastfeeding as the primary independent variable of interest, adjusted for the following confounders: age, BMI, parity, and intervention. The models for all women (n=485) were also adjusted for ethnicity.

Supplementary Figure 2a: Standard deviation differences in fatty acids, amino acids, glycaemic and other markers between breastfeeding and non-breastfeeding UPBEAT women at 6 months postpartum [for all women=485, those of White ethnicity (n=356) and those of Black ethnicity (n=83)]. The right-hand side of the x-axis represents positive associations with breastfeeding (fully or mixed) at 6 months, compared to non-breastfeeding women, and negative associations to the left-hand side. Multivariable linear regression was applied for each metabolite with breastfeeding as the primary independent variable of interest, adjusted for the following confounders: age, BMI, parity, and intervention. The models for all women (n=485) were also adjusted for ethnicity.

Supplementary Figure 2b: Standard deviation differences in lipoprotein particle groups between breastfeeding and non-breastfeeding UPBEAT women at 6 months postpartum [for all women=485, those of White ethnicity (n=356) and those of Black ethnicity (n=83)]. The right-hand side of the x-axis represents positive associations with breastfeeding (fully or mixed) at 6 months, compared to non-breastfeeding women, and negative associations to the left-hand side. Multivariable linear regression was applied for each metabolite with breastfeeding as the primary independent variable of interest, adjusted for the following confounders: age, BMI, parity, and intervention. The models for all women (n=485) were also adjusted for ethnicity.

a

b

Supplementary figures 3a and 3b: Standard deviation difference in lipoprotein particle concentration and subclass constituents between breastfeeding and non-breastfeeding UPBEAT women at 6 months postpartum in the control arm only (n=253). The right-hand side of the x-axis represents positive associations with breastfeeding (fully or mixed) at 6 months, compared to non-breastfeeding women, and negative associations to the left-hand side. Multivariable linear regression was applied for each metabolite with breastfeeding as the primary independent variable of interest, adjusted for the following confounders: age, BMI, parity, and intervention. The models for all women (n=485) were also adjusted for ethnicity.

a

b

Supplementary figures 4a and 4b: Standard deviation differences for (a) fatty acids, amino acids, glycaemic and other markers and (b) lipoprotein particle groups between breastfeeding and non-breastfeeding UPBEAT women at 6 months postpartum in the control arm only (n=253). The right-hand side of the x-axis represents positive associations with breastfeeding (fully or mixed) at 6 months, compared to non-breastfeeding women, and negative associations to the left-hand side. Multivariable linear regression was applied for each metabolite with breastfeeding as the primary independent variable of interest, adjusted for the following confounders: age, BMI, parity, and intervention. The models for all women (n=485) were also adjusted for ethnicity.
